# An Approach to Macroscopic Central Lymph Nodes Detected during Surgery in Patients with Thyroid Micropapillary Carcinoma: Should We Resort to Dissection?

**DOI:** 10.1155/2017/5814610

**Published:** 2017-02-23

**Authors:** Osman Kurukahvecioglu, Kursat Dikmen, Hasan Bostanci, Murat Akin, Ferit Taneri

**Affiliations:** Department of General Surgery, Faculty of Medicine, Gazi University, Ankara, Turkey

## Abstract

*Background*. High-resolution ultrasonography and the ability to perform fine-needle aspiration biopsy even for nodules smaller than three millimeters have considerably increased the detection rate of thyroid micropapillary carcinoma (TMPC). Despite favorable prognosis, the prevalence of cervical lymph node metastases in patients with TMPC is approximately 30%. *Aim.* In this study, we aimed to determine the central lymph node metastasis rate and its relation to the characteristics of the tumor. *Methods.* One hundred nine patients who underwent surgery due to TMPC between December 2009 and January 2014 were analyzed retrospectively. Patients were divided into two groups according to whether they underwent lymph node dissection and the two groups were then compared with respect to tumor size and multicentricity, age, and presence of lymphocytic thyroiditis. *Results.* There were no statistically significant differences between the two groups of patients in terms of tumor size, tumor multicentricity, age, and presence of lymphocytic thyroiditis. When the patient group that received lymph node dissection was further analyzed, it was found that patients with lymphocytic thyroiditis had a significantly lower number of metastatic lymph nodes. *Conclusion.* Central lymph node dissection in TMPC patients with macroscopic lymph node detected intraoperatively would ensure accurate staging without an increase in morbidity.

## 1. Introduction

Thyroid cancer is the most common cancer of the endocrine system accounting for approximately 1% of all cancers and 30–35% of head and neck cancers. Papillary thyroid carcinoma constitutes 80–85% of thyroid carcinomas and its ten-year survival rate is more than 90% [1]. According to the World Health Organization (WHO), the definition “thyroid micropapillary carcinoma (TMPC)” is used if the tumor size is less than one centimeter in diameter. As the size suggests, TMPC can be easily missed during clinical evaluation [2, 3].

While in the past TMPC was mostly found in the surgical specimens of thyroidectomies due to a benign disease and in the autopsy series, today the detection rate of TMPC has considerably increased due to the widespread use of high-resolution ultrasonography (USG) and fine-needle aspiration biopsy for nodules smaller than three millimeters [4, 5]. Despite favorable prognosis, the rate of cervical lymph node metastasis (CLNM) found in the specimens of the patients with TPMC is around 30–60% [6–8]. Central lymph node dissection (CLND) is generally recommended for cases with a high rate of local recurrence [9]. However, for TMPC patients, who do not show any clinically or radiologically detectable lymph nodes and are staged as cN0 (clinical node stage 0), prophylactic CLND remains controversial. The aim of this study is to determine the central lymph node metastasis rate in TMPC and its relation to the characteristics of the tumor.

## 2. Materials and Methods

One hundred nine patients operated with the diagnosis of papillary thyroid carcinoma between December 2009 and January 2014 were included in this study. The inclusion criteria were as follows: (1) tumor size less than or equal to one centimeter, measured via USG, (2) no clinical or radiological evidence of cervical lymphadenopathy (cN0), (3) younger than 80 years of age, (4) presence of more than six lymph nodes in the CLND specimen (if CLND was performed), and (5) no prior history of head and neck radiation or surgery. For multicentric tumors, one-centimeter criteria were applied to the single biggest tumor. Preoperative diagnosis was made in all patients via cytological evaluation of fine-needle aspiration biopsy (FNAB). Although FNAB is not generally required for tumors less than one centimeter in size, all our patients showed suspicious findings on USG such as irregular nodule surface or increased vascularity. The patients were evaluated both clinically and with USG and staged as cN0. We divided the patients into two groups according to the surgical procedure performed. The first group was the patients who underwent only total thyroidectomy (*n* = 53), and the second group was the patients who underwent total thyroidectomy and CLND (*n* = 56). The indication for unilateral or bilateral CLND was determined intraoperatively upon finding of macroscopic lymph nodes in the central neck region. CLND includes prelaryngeal, paralaryngeal, pretracheal, paratracheal, periglandular, and paraglandular lymph nodes between the hyoid bone and level 7. Also partial thymectomy was performed to all patients. All of the patients were operated by the same team experienced in endocrine surgery. Surgical specimens were also evaluated by the same pathologist.

The two groups were compared in terms of age, gender, tumor multicentricity, tumor size, and presence of lymphocytic thyroiditis. The CLND group was also further evaluated for lymph node metastasis.

We did not observe any morbidity related to hypocalcemia or recurrent nerve damage in the patients who has undergone CLND. All specimens were carefully evaluated for any presence of parathyroid tissue at the operating table before being sent to pathology.

SPSS 17.0 software was used for statistical evaluation. Intergroup evaluation was done using *t*-test, chi-square test, the Mann–Whitney *U* test, and Fisher's exact test; *p* < 0.05 was considered as statistically significant.

## 3. Results

Descriptive statistics of the patients included in this study are given in Table 1. One hundred nine patients (median age: 43 y (range: 19–79 y), 79 females) were operated with the diagnosis of papillary thyroid carcinoma. Average tumor size was 7.34 ± 2.53 mm, and the tumor was multifocal in 19.3% of the cases. Lymphocytic thyroiditis was present in 41.3% of the patients. Among the patients who underwent CLND (*n* = 56), metastasis was detected in 13 patients (23.2%).

No statistically significant difference was found between the two study groups (total thyroidectomy versus total thyroidectomy + CLND) in terms of age, gender, tumor multicentricity, lymphocytic thyroiditis, tumor size, and number of nodules (Table 2).

The patients who received CLND as a part of their treatment are further analyzed by dividing this group into two subgroups according to metastatic status (absent/present). No statistically significant differences were found upon the comparisons of the two subgroups in terms of age, gender, tumor multicentricity, tumor size, number of nodules, and number of lymph nodes removed (Table 3). Patients with lymphocytic thyroiditis were found to have significantly fewer metastatic lymph nodes (Table 3) (*p* < 0.05).

## 4. Discussion

Papillary thyroid cancers with a diameter of less than ten millimeters are called thyroid micropapillary carcinoma (TMPC). In the recent years, the incidence of thyroid micropapillary carcinoma has increased [4, 10]. TMPC has been mainly reported on autopsy studies before the widespread use of advanced ultrasound equipment. Though encountered randomly after thyroid surgery, it was reported to have an incidence rate of 2–24% [11]. Although TMPC is accepted to have good prognosis, the reported rates of mortality, lymph node metastasis, and distant metastasis are 1%, 5%, and 2.5%, respectively [12]. In a study carried out at the Mayo Clinic, multicentricity, lymph node metastasis, and distant metastases were found to be 23%, 30%, and 0.3%, respectively [13]. For an average of ten-year follow-up, recurrence was reported in 3.5% and distant metastasis in 0.2% of the patients [13], and the majority of the patients with recurrence were found to be in the form of lymph node metastases [13]. Lymph node metastasis rates appear to be associated with the tumor size, especially tumors over five millimeters which were reported to exhibit greater probability of lymph node metastases [12]. In addition, lower rates of recurrence were shown in connection to presence of thyroid autoimmunity together with microcarcinoma.

Neck lymph node metastasis in TMPC was reported to be approximately 24% to central lymph nodes and 3.7% to lateral group lymph nodes [14]. The central region is the first and most frequent area of thyroid carcinoma metastasis and constitutes one of the most important prognostic markers for recurrence [15, 16]. In line with previous studies, we have found central neck lymph node metastasis in 23% of the cases that underwent neck dissection. While clinically detected lymph node recurrence rate is between 15 and 50% in patients with papillary carcinoma, occult metastasis rate is between 40 and 90% [2, 17, 18, 19]. Although this rate is lower in micropapillary carcinomas, it is still at a considerable level of 20–40% [7, 19, 20]. Despite the importance of lymph node metastases, dissection of central group lymph nodes during thyroidectomy is still under debate because of possible morbidities such as hypoparathyroidism and recurrent nerve injury or perhaps because of the belief that survival rate is not affected by lymph node metastasis [21, 22]. Although lateral or central neck dissection is performed routinely in addition to total thyroidectomy when metastasis to cervical lymph nodes is known, prophylactic neck dissection to cN0 patients is still controversial. The effect of prophylactic neck dissection on recurrence rates for papillary carcinomas appears to be negligible or substantial [23, 24]. Contradicting results might be due to inhomogeneous population with respect to risk factors. It appears that the most important reason why prophylactic neck dissection has not gained wide acceptance is the morbidity of the procedure itself. However, in experienced hands, CLND can be performed without any additional morbidity to that of a total thyroidectomy. Today, total thyroidectomy complications such as damage to the laryngeal nerve or parathyroid glands are rare as the surgeons are experienced and/or specialized in thyroid surgery. Similarly, experience in neck dissection would minimize the complications and morbidity of the procedure. The relatively benign course of papillary thyroid carcinoma has led to a view that CLND would not affect survival rates. This view, however, has not been supported yet by any long-term prospective studies. Our study, although with a limited number of cases, shows that the morbidity of CLND can be minimized to that of a total thyroidectomy.

Some authors argue that in case of intraoperative observation or palpation of lymph nodes in cN0 patients, the patients should be restaged as N+ and central neck dissection should be performed. However, it is emphasized that CLND should be performed by experienced hands [25, 26]. On the other hand, some authors are against the prophylactic dissection and claim that it would increase morbidity. Some would even argue that in case of central neck metastases, there would also be metastases at level seven (L7); thus CLND would not be the optimum treatment [18, 19]. In this study, we evaluated the patients who were staged as cN0 preoperatively during surgery and upon finding any visible/palpable lymph nodes, we restaged them as N+ and performed CNLD. We aimed to investigate the central lymph node metastasis rate in TMPC in order to provide information to whether a prophylactic neck dissection can be justified for cN0 TMPC patients.

In countries with endemic thyroiditis, enlarged lymph nodes are often observed in the central neck region and preoperative differentiation of metastatic or reactive lymph nodes is challenging. In this study, we observed that more than half of the patients who underwent CLND (26 out of 56) had lymphocytic thyroiditis, which would be expected to present with enlarged lymph nodes. However, by means of CNLD, we were able to detect the patients who had concurrent lymph node metastasis (two out of 26).

It has been claimed that both low thyroglobulin levels and low recurrence rates would be obtained by performing CLND at the primary operation and thus complications due to a secondary surgical intervention would decrease [20]. Evaluation of the CLND specimen advances the disease stage in 30% of the patients [27, 28]. Therefore, one might argue CLND to be an important part of the first line of treatment that would improve the accuracy of staging and would affect the survival rate in long term [27, 28]. Decrease in thyroglobulin (TG) and antithyroglobulin (anti-TG) values which are used in the postoperative follow-up of these patients is one of the most important indicators of possible lymph node metastasis.

In the postoperative follow-up, the thyroglobulin and antithyroglobulin values are important markers for lymph node metastasis and are useful in planning radioactive iodine (RAI) treatment. Complete removal of tumor would ensure the lowest levels of these markers and optimize their use in the follow-up.

Various parameters are utilized while evaluating the prognosis of papillary thyroid carcinoma and the most commonly used parameters are age, gender, family history, tumor size, presence of extracapsular extension, and presence of metastasis. Although these parameters provide insight to the prognosis of disease, the most accurate evaluation is done through histopathological evaluation. Moreover, the prognostics factors do not include central lymph node metastasis. As recurrence is most commonly seen in the central neck, it might indicate inadequate surgery [29]. CLND would help minimize recurrence or persistent disease in TMPC patients with central lymph node metastasis. The American Thyroid Association (ATA) guideline [9] places the patients with central lymph node metastasis to moderate risk group and recommends RAI treatment for this patient group. As CLND provides the exact status of neck (N0 or N+), it gives us the essential information for deciding whether or not to proceed with RAI treatment.

We advocate central group lymph node dissection when macroscopic lymph nodes are observed during the intraoperative evaluation of the patients with thyroid micropapillary carcinoma. If the tumor is unifocal, ipsilateral lymph nodes are dissected while if the tumor is multifocal, bilateral central lymph nodes are dissected. Since our team is experienced in this field, central lymph node dissections were performed with morbidity rates equivalent to total thyroidectomy. The actual disease stage was then determined by the evaluation of the lymph nodes in the surgical specimen. Although one might argue that this would not affect the prognosis, the additional information on the lymph node status of the patient would be useful in the follow-up and further planning of the treatment. In addition, performing dissection during the primary surgical treatment would prevent disease recurrence. Secondary benefits would be minimizing patients' anxiety due to the possibility of a secondary operation and preventing morbidity due to secondary interventions.

In patients with lymph node metastases, the common feature of the metastases is being in the form of micrometastases. Tumor multicentricity, lymph node metastases, and distant metastases have been reported to be 20%–40%, 17%–43%, and 0.3%, respectively [15, 16]. In our study, 23% of the CLND patients were found to have metastatic lymph nodes. The CLND should be viewed as a staging procedure with therapeutic value. In the hands of experts, the morbidity is not different from total thyroidectomy with an additional benefit of preventing local recurrences. Accurate staging of thyroid micropapillary carcinoma by means of CLND would improve both the evaluation of prognosis and postoperative follow-up.

## Figures and Tables

**Table 1:** Descriptive statistics of the patients (*N* = 109).

	Number (percentage)
Gender	
Female	79 (72.5%)
Male	30 (27.5%)

Multicentricity	
No	88 (80.7%)
Yes	21 (19.3%)

Surgery	
Total thyroidectomy + CLND	56 (51.4%)
Total thyroidectomy	53 (48.6%)

Lymphocytic thyroiditis	
Absent	64 (58.7%)
Present	45 (41.3%)

**Table 2:** Comparison of age, gender, and multicentricity; presence of lymphocytic thyroiditis; tumor size; and number of nodules in the two patient groups.

	Patient group		
	Total thyroidectomy (*n* = 53)	Total thyroidectomy + CLND (*n* = 56)	
Age (median, range)	41.5 y (25–69 y)	44.0 y (19–79 y)	*p* = 0.53^∗^

Gender			
Female (*n*, %)	39 (49.4%)	40 (50.6%)	*p* = 0.97^∗∗^
Male (*n*, %)	14 (46.7%)	16 (53.3%)	

Multicentricity			
No (*n*, %)	45 (51.1%)	43 (48.9%)	*p* = 0.40^∗∗^
Yes (*n*, %)	8 (38.1%)	13 (61.9%)	

Lymphocytic thyroiditis			
Absent (*n*, %)	34 (53.1%)	30 (46.9%)	*p* = 0.35^∗∗^
Present (*n*, %)	19 (42.2%)	26 (57.8%)	

Tumor size (median, range)	8.0 (2–10) mm	8.0 (2–10) mm	*p* = 0.32^∗∗∗^

Number of nodules (median, range)	1.0 (1–5)	1.0 (1‐2)	*p* = 0.22^∗∗∗^

^∗^ *p* values obtained from independent sample *t*-test; ^∗∗^*p* values obtained from Yate's corrected chi-square test; ^∗∗∗^*p* values obtained from the Mann–Whitney *U* test.

**Table 3:** Further analyses of the patient group who underwent total thyroidectomy + CLND (*N* = 56). Patients are subdivided into two groups according to metastatic status in the CNLD specimen. Comparison of age, gender, and tumor multicentricity; presence of lymphocytic thyroiditis; tumor size; number of nodules; and the number of lymph nodes removed was performed between the groups.

	Metastases		
	Absent (*N* = 43)	Present (*N* = 13)	
Age (median, range)	42.0 y (25–60 y)	37.0 y (27–69 y)	*p* = 0.47^∗^

Gender			
Female (*n*, %)	31 (77.5%)	9 (22.5%)	*p* = 1.00^∗∗^
Male (*n*, %)	12 (75.0%)	4 (25.0%)	

Multicentricity			
No (*n*, %)	34 (79.1%)	9 (20.9%)	*p* = 0.47^∗∗^
Yes (*n*, %)	9 (69.2%)	4 (30.8%)	

Lymphocytic thyroiditis			
Absent (*n*, %)	19 (63.3%)	11 (36.7%)	*p* = 0.02^∗∗^
Present (*n*, %)	24 (92.3%)	2 (7.7%)	

Tumor size (median, range)	8 (2–10) mm	9 (5–10) mm	*p* = 0.24^∗^

Number of nodules (median, range)	1.0 (1–5)	1 (1‐2)	*p* = 0.60^∗^

Number of lymph nodes removed (median, range)	6 (1–6)	7 (6–17)	*p* = 0.60^∗^

^∗^ *p* values obtained from the Mann–Whitney *U* test; ^∗∗^*p* values obtained from Fisher's exact test.
